# Data mining of acupoint selection and combination patterns for dysphagia with sialorrhea: a literature-based study

**DOI:** 10.3389/fneur.2026.1849484

**Published:** 2026-09-22

**Authors:** Haihan Zhang, Yizhou Liu, Shikay Loong, Shangyu Wei, Yuze Wang, Wei Li, Yu Feng, Weidong Pan

**Affiliations:** 1 Department of Neurology, Shuguang Hospital Affiliated to Shanghai University of Traditional Chinese Medicine, Shanghai, China; 2 Center of Neurology and Neurorehabilitation, Shanghai Yangzhi Rehabilitation Hospital (Shanghai Sunshine Rehabilitation Center), School of Medicine, Tongji University, Shanghai, China; 3 Shanghai Pudong New Area Guangming Traditional Chinese Medicine Hospital, Shanghai, China; 4 First Department of Encephalopathy, Anhui Branch of Shuguang Hospital Affiliated to Shanghai University of Traditional Chinese Medicine, Hefei, Anhui, China

**Keywords:** acupuncture, data mining, dysphagia, excessive salivation, sialorrhea

## Abstract

**Background:**

Dysphagia accompanied by sialorrhea/drooling is clinically challenging, and acupuncture is frequently applied; however, standardized summaries of acupoint selection and combination patterns remain limited.

**Methods:**

Eight databases were searched from inception to May 2025. Clinical publications reporting acupuncture prescriptions for dysphagia with sialorrhea/drooling were included. One representative prescription was extracted from each eligible article (124 articles; 124 prescriptions). Acupoint names were normalized according to the GB/T standard terminology. Descriptive statistics were used to summarize acupoint frequency, meridian distribution, and commonly used acupoint categories. Association rule mining was performed using the Apriori algorithm (SPSS Modeler 18.0), and a co-occurrence network was visualized in Cytoscape to identify core nodes and stable combinations.

**Results:**

A total of 124 prescriptions containing 179 unique acupoints were extracted (1,070 total acupoint occurrences). The most frequently used acupoints included CV23, GB20, EX-HN12, EX-HN13, GV20, LI4, and ST36. Association rules and network analysis revealed stable co-occurrence structures, highlighting several high-connectivity acupoints and recurring pairs/sets (e.g., CV23–GB20; EX-HN12–EX-HN13; ST4–ST6, where applicable).

**Conclusion:**

Literature-based prescription data mining identified commonly used acupoints and reproducible co-occurrence patterns for dysphagia with sialorrhea. These findings provide a quantitative basis for acupoint selection and may inform the development of standardized clinical protocols, as well as future prospective validation.

## 1 Introduction

Dysphagia, otherwise referred to as a swallowing disorder, is a prevalent symptom that has the capacity to significantly reduce the quality of life experienced by patients. It is estimated that 590 million people worldwide are affected by dysphagia, a condition caused by various diseases (1). Research indicates that dysphagia may affect 10 to 33% of older adults, particularly those with a history of neurological disorders or esophageal diseases (2). Dysphagia may lead to increased salivation, aspiration pneumonia, nutritional disorders. Among these, drooling is a common occurrence (3). The primary cause of drooling due to dysphagia is impaired swallowing mechanisms, preventing effective clearance of saliva. In dysphagia patients, increased drooling typically stems not from an absolute rise in saliva production but from diminished clearance capacity (4). Sialorrhea in dysphagic patients results from the combined impairment of salivary clearance and central neurologic control over secretion and swallowing. Based on the course of the disease, dysphagia may be acute or chronic, intermittent or persistent. Based on the cause, dysphagia can be classified into neuromuscular, structural, infectious, and inflammatory causes (5).

The treatment methods vary for different primary diseases. For swallowing disorders accompanied by sialorrhea caused by cerebrovascular accidents, rehabilitation training is emphasized during the acute and recovery phases. In the case of diseases such as Parkinson’s disease, the focus of treatment is primarily on improving the underlying disease. For tumors and obstructive esophageal diseases, the treatment priority is to relieve obstruction, primarily through surgical intervention or radiotherapy. Controlling secretions while avoiding dry mouth and gastrointestinal problems is a challenging task for healthcare professionals. Drug therapies carry certain side effects, while behavioral and physical rehabilitation treatments rely heavily on physical equipment. Therefore, seeking diverse, effective, and convenient treatment approaches for dysphagia is crucial for improving patients’ quality of life.

Research has shown that acupuncture, electroacupuncture, and the combination of acupuncture with other therapies can effectively alleviate symptoms of dysphagia and excessive salivation (6). Acupuncture therapy demonstrates positive effects for both pseudobulbar palsy caused by upper motor neuron damage and true bulbar palsy resulting from medullary lesions. It effectively alleviates functional impairments such as dysphagia and drooling (7, 8).

Currently, there is no systematic review focusing on the clinical application of acupuncture for the treatment of dysphagia combined with sialorrhea. Randomized controlled trials and meta-analyses have indicated that acupuncture can effectively improve symptoms in patients with dysphagia (9, 10). Data mining refers to the process of exploring underlying patterns and regularities from large datasets through specific algorithms to uncover potential application value. Its core significance lies in transforming raw data into actionable knowledge. This study aims to utilize data mining techniques to analyze the principles of acupoint selection and combination documented in the literature on acupuncture for dysphagia accompanied by sialorrhea, thereby providing a scientific basis for acupoint choice in the clinical management of patients with dysphagia.

## 2 Methods

### 2.1 Literature search

A systematic literature search was conducted up to May 2025 using the Shanghai University of Traditional Chinese Medicine library platform to retrieve relevant studies from multiple databases, including CNKI, CQVIP, Wanfang, PubMed, SinoMed, Web of Science, ScienceDirect, Cochrane Library. The search strategy incorporated Medical Subject Headings (MeSH) and keywords such as “acupuncture,” “acupoint,” “meridian,” “fire needling,” “swallowing,” “dysphagia,” “salivation,” and “hypersalivation.” The search syntax was tailored to the specific requirements of each database. To minimize the risk of omitting relevant records, a manual search of reference lists was also performed to ensure comprehensive coverage.

### 2.2 Inclusion and exclusion criteria

Inclusion Criteria: (1) study type: eligible clinical studies included randomized or non-randomized controlled studies, cohort studies, and before–after studies that enrolled more than one patient, reported group-level clinical data, and provided a reproducible acupuncture prescription; (2) subjects of research: patients diagnosed with dysphagia and comorbid hypersalivation, including those with true or pseudobulbar palsy resulting from conditions such as stroke, Parkinson’s disease, and amyotrophic lateral sclerosis; (3) interventions: the therapeutic approaches were defined as follows: the experimental group underwent interventions centered on acupuncture, moxibustion, or electroacupuncture, either independently or integrated with adjunctive therapies. The intervention measures in the control group include different acupuncture therapies, Chinese medicine therapies, Western medicine therapies, placebo control, etc.

Exclusion criteria: (1) Duplicate publications; (2) Single-patient case reports; (3) Narrative expert-experience reports or descriptive summaries without group-level clinical data; (4) Reviews, systematic reviews, and meta-analyses; (5) Animal or other preclinical studies; (6) Conference abstracts, protocols, commentaries, and letters; (7) Reports for which the full text was unavailable.

### 2.3 Data extraction and processing

The study methodology comprised the following steps: (1) Preliminary screening of retrieved records by title and abstract to exclude duplicates and irrelevant studies; (2) Full-text assessment of potentially eligible articles against the inclusion and exclusion criteria, with exclusion of unavailable full texts; (3) Two reviewers independently extracted the data using a standardized extraction form, and disagreements were resolved through discussion or consultation with a third reviewer.; (4) Entry of acupoint data into a Microsoft Excel 2019 database, standardized according to Chinese National Standards GB/T 12346-2021 and GB/T 40997-2021 (11, 12). For studies reporting multiple acupuncture groups, the prescription from the experimental group (defined as the group receiving acupuncture as the primary or adjunctive intervention) was extracted. For studies using a fixed core prescription with optional adjunct points, all points listed in the core prescription were included; adjunct points that were explicitly described as “added based on syndrome differentiation” were not included unless they appeared in ≥50% of the cases within that study; (5) Cross-verification of all data entries; (6) Association rule mining was performed using the Apriori algorithm in SPSS Modeler 18.0. For descriptive analysis, all recorded acupoint occurrences were counted. For association-rule analysis, each acupoint was coded as present or absent within each prescription, and repeated records within the same prescription were counted once. The minimum antecedent support and confidence thresholds were set at 10 and 95%, respectively, and the maximum antecedent size was set at two acupoints; (7) the core acupoint associations were visualized using Cytoscape 3.9.1. The core acupoint associations identified through rule analysis were visualized using Cytoscape 3.9.1 to illustrate the prescription network.

## 3 Results of data mining

### 3.1 Research content

This study initially performed a preliminary search across eight databases, identifying a total of 1,515 articles, distributed as follows: CNKI (705 articles), CQVIP (26 articles), Wanfang (104 articles), PubMed (236 articles), Sinomed (64 articles), Web of Science (271 articles), ScienceDirect (107 articles), and Cochrane Library (2 articles). After removing duplicates, the number of articles was reduced to 1,197. The process of exclusion and selection is illustrated in Figure 1.

**Figure 1:**
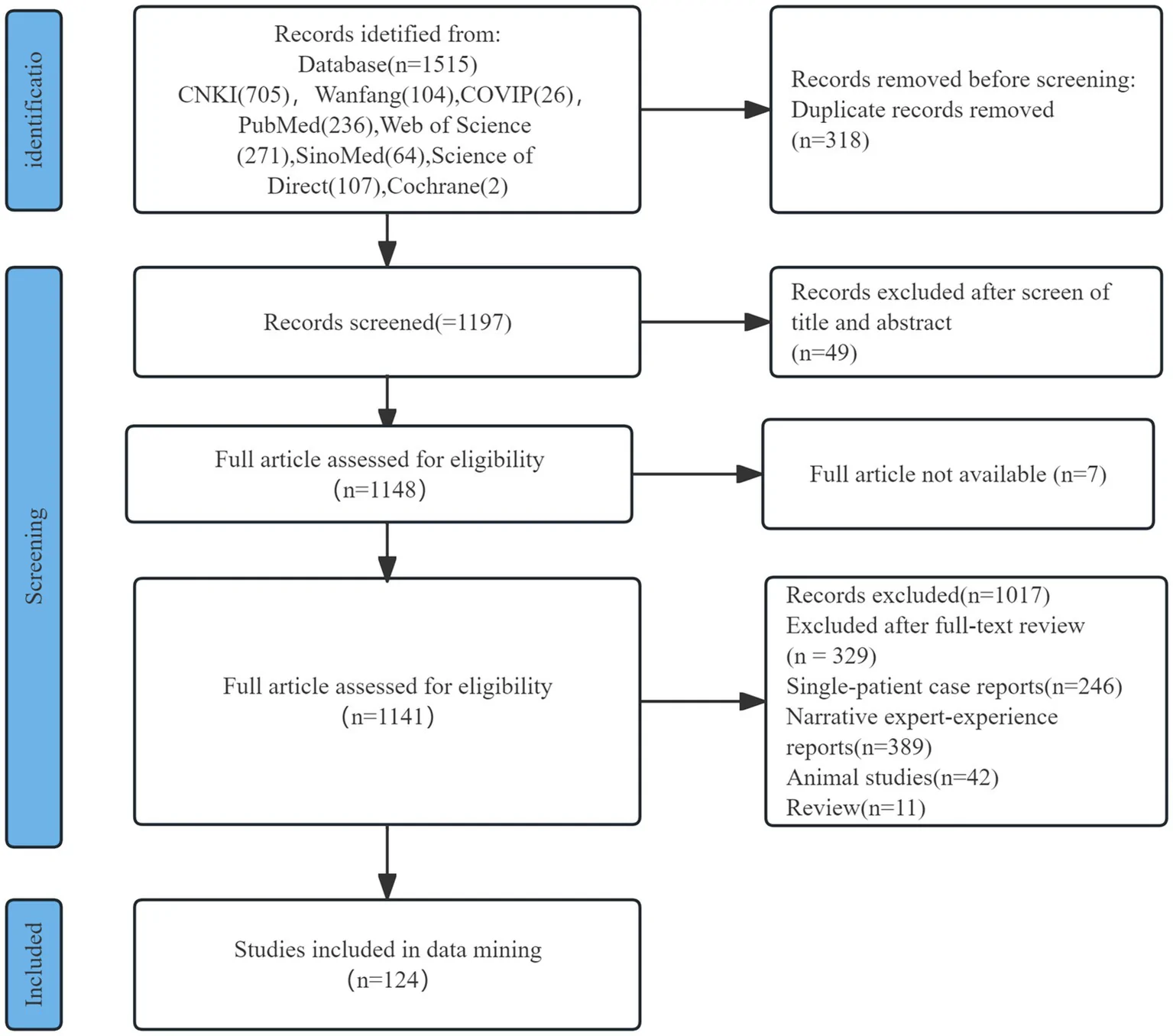
Flowchart of the study selection process.

### 3.2 Descriptive analysis

#### 3.2.1 Analysis of the frequency of acupoint usage

This study included a total of 124 studies, covering 179 acupuncture points with a total frequency of 1,070 occurrences. The five most frequently used acupuncture points were as follows: Lianquan (CV23) (82 times, 66.13%), Fengchi (GB20) (59 times, 47.58%), Jinjin (EX-HN12) (42 times, 33.87%), Yuye (EX-HN13) (42 times, 33.87%), and Baihui (GV20) (38 times, 30.65%). Figure 2 illustrates the ranking of the top thirty most frequently utilized acupoints.

**Figure 2:**
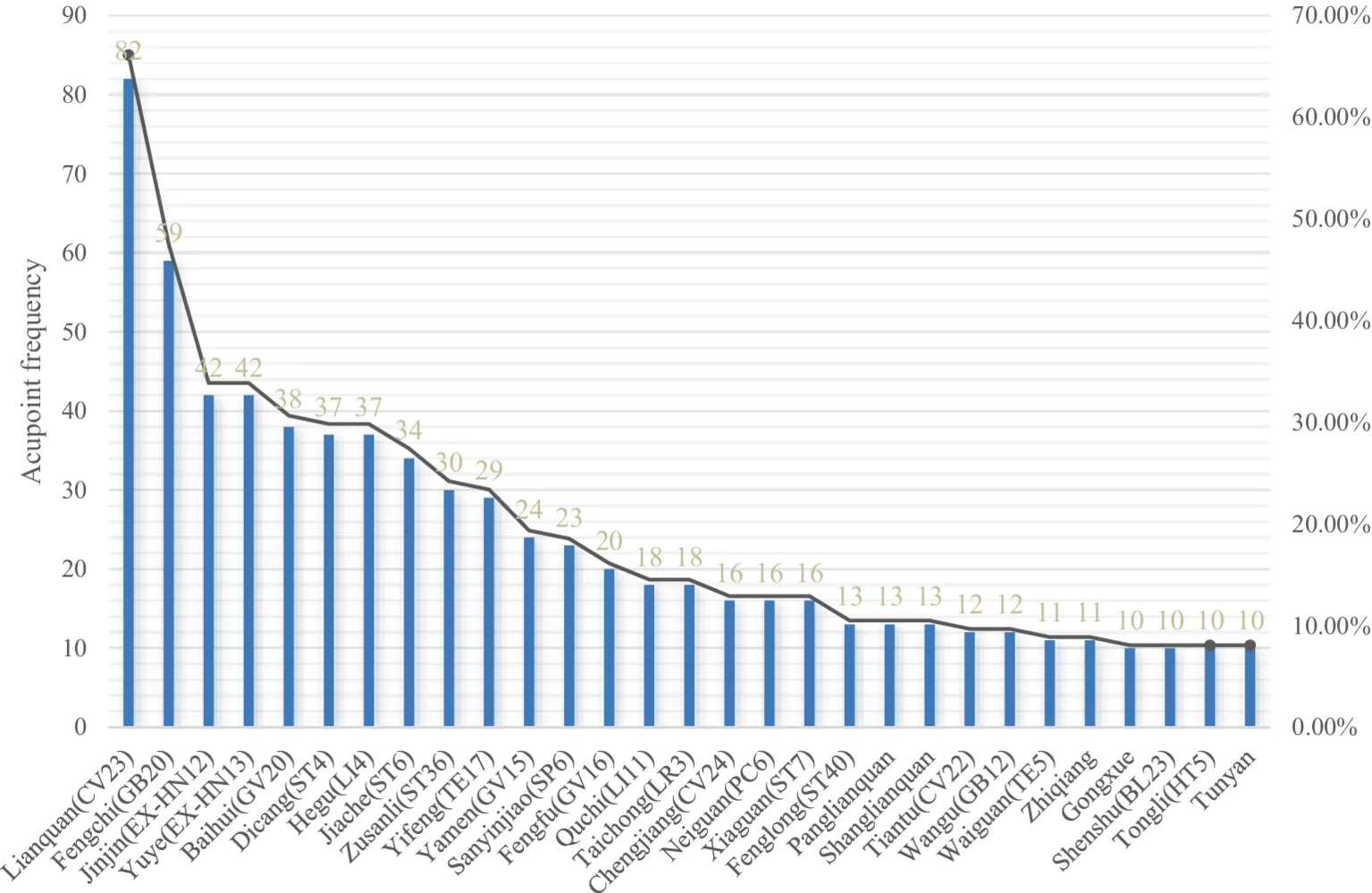
Top 30 most frequently utilized acupoints for treating dysphagia with excessive salivation.

#### 3.2.2 Analysis of meridian attribution of acupoints

The meridian most frequently involved was the Foot Yangming Stomach Meridian (ST, 154 times), followed by the Conception Vessel (CV, 125 times), Governing Vessel (GV, 123 times), and the Foot Shaoyang Gallbladder Meridian (GB, 104 times). These four meridians together accounted for 56 acupuncture points, representing 31.3% of the total acupuncture points. The most frequently used acupuncture points were Lianquan (CV23) (82 occurrences, 66.13%) and Fengchi (GB20) (59 occurrences, 47.58%). Following these, Jinjin (EX-HN12) and Yuye (EX-HN13) were recorded 42 times each (33.87%), as extra points. The meridian distribution and high-frequency acupuncture points are shown in Tables 1, 2.

**Table 1:** Meridian attribution of acupuncture points in dysphagia with sialorrhea treatment.

Meridians	Frequency	Acupoint number	Acupoints (frequency)
ST	154	16	Dicang (ST4) (37), Jiache (ST6) (34), Zusanli (ST36) (30), Xiaguan (ST7) (16), Fenglong (ST40) (13), Renying (ST9) (9), Daying (ST5) (4), Liangqiu (ST34) (2), Jiexi (ST41) (2), Touwei (ST8) (1), Tianshu (ST25) (1), Guilai (ST29) (1), Biguan (ST31) (1), Futu (ST32) (1), Chongyang (ST42) (1), Sibai (ST2) (1)
CV	125	8	Lianquan (CV23) (82), Chengjiang (CV24) (16), Tiantu (CV22) (12), Zhongwan (CV12) (6), Guanyuan (CV4) (5), Qihai (CV6) (2), Danzhong (CV17) (1), Huagai (CV20) (1)
GV	123	17	Baihui (GV20) (38), Yamen (GV15) (24), Fengfu (GV16) (20), Shuigou (GV26) (9), Shangxing (GV23) (8), Shenting (GV24) (6), Dazhui (GV14) (4), Mingmen (GV4) (3), Shenzhu (GV12) (2), Naohu (GV17) (2), Yaoyangguan (GV3) (1), Xuanshu (GV5) (1), Jizhong (GV6) (1), Zhongshu (GV7) (1), Lingtai (GV10) (1), Shendao (GV11) (1), Taodao (GV13) (1)
GB	104	15	Fengchi (GB20) (59), Wangu (GB12) (12), Yanglingquan (GB34) (9), Huantiao (GB30) (5), Xuanzhong (GB39) (4), Benshen (GB13) (2), Toulinqi (GB15) (2), Fengshi (GB31) (2), Zulinqi (GB41) (2), Xiaxi (GB43) (2), Shuaigu (GB8) (1), Yangbai (GB14) (1), Naokong (GB19) (1), Jianjing (GB21) (1), Qiuxu (GB40) (1)
LI	74	7	Hegu (LI4) (37), Quchi (LI11) (18), Jianyu (LI15) (8), Shousanli (LI10) (5), Futu (LI18) (3), Shangyang (LI1) (2), Yingxiang (LI20) (1)
TE	47	7	Yifeng (TE17) (29), Waiguan (TE5) (11), Zhigou (TE6) (2), Jianliao (TE14) (2), Zhongzhu (TE3) (1), Yangchi (TE4) (1), Sizhukong (TE23) (1)
BL	42	15	Shenshu (BL23) (10), Tianzhu (BL10) (6), Xinshu (BL15) (5), Pishu (BL20) (4), Feishu (BL13) (2), Geshu (BL17) (2), Weizhong (BL40) (2), Chengshan (BL57) (2), Kunlun (BL60) (2), Cuanzhu (BL2) (1), Tongtian (BL7) (1), Fengmen (BL12) (1), Weishu (BL21) (1), Dachangshu (BL25) (1), Ganshu (BL18) (2)
SP	30	5	Sanyinjiao (SP6) (23), Yinlingquan (SP9) (3), Taibai (SP3) (2), Gongsun (SP4) (1), Diji (SP8) (1)
LR	20	2	Taichong (LR3) (18), Xingjian (LR2) (2)
KI	19	4	Zhaohai (KI6) (9), Taixi (KI3) (7), Yongquan (KI1) (2), Jiaoxin (KI8) (1)
PC	17	2	Neiguan (PC6) (16), Daling (PC7) (1)
SI	16	6	Tianrong (SI17) (4), Jianzhen (SI9) (4), Houxi (SI3) (2), Tianzong (SI11) (2), Tianchuang (SI16) (2), Quanliao (SI18) (2)
HT	15	3	Tongli (HT5) (10), Shenmen (HT7) (4), Shaofu (HT8) (1)
LU	13	5	Lieque (LU7) (5), Chize (LU5) (3)，Taiyuan (LU9) (2), Yuji (LU10) (2), Shaoshang (LU11) (1)

**Table 2:** Frequency and percentage of acupoints used for dysphagia with excessive salivation.

Specific point		Acupoint frequency		Acupoint number		Acupoints (frequency)
		Frequency	Percentage	Number	Percentage	
Five-shu point	Jing-well point	5	0.47%	3	1.68%	Shangyang (LI1) (2), Yongquan (KI1) (2), Shaoshang (LU11) (1)
	Ying-spring point	7	0.65%	4	2.23%	Xiaxi (GB43) (2), Xingjian (LR2) (2), Yuji (LU10) (2), Shaofu (HT8) (1)
	Shu-stream point	39	3.64%	9	5.03%	Taichong (LR3) (18), Taixi (KI3) (7), Shenmen (HT7) (4), Houxi (SI3) (2), Taibai (SP3) (2), Taiyuan (LU9) (2), Zulinqi (GB41) (2), Daling (PC7) (1)， Zhongzhu (TE3) (1)
	Jing-river point	6	0.56%	3	1.68%	Jiexi (ST41) (2), Kunlun (BL60) (2), Zhigou (TE6) (2)
	He-sea point	65	6.07%	6	3.35%	Zusanli (ST36) (30), Quchi (LI11) (18), Yanglingquan (GB34) (9), Chize (LU5) (3), Yinlingquan (SP9) (3), Weizhong (BL40) (2)
Total		122	11.40%	25	13.97%	
Yuan-primary point		74	6.92%	10	5.59%	Hegu (LI4) (37), Taichong (LR3) (18), Taixi (KI3) (7), Shenmen (HT7) (4), Taibai (SP3) (2), Taiyuan (LU9) (2), Chongyang (ST42) (1), Daling (PC7) (1), Qiuxu (GB40) (1), Yangchi (TE4) (1)
Luo-connecting point		56	5.23%	6	3.35%	Neiguan (PC6) (16), Fenglong (ST40) (13), Waiguan (TE5) (11), Tongli (HT5) (10), Lieque (LU7) (5), Gongsun (SP4) (1)
Eight confluence points		46	4.30%	7	3.91%	Neiguan (PC6) (16), Waiguan (TE5) (11), Zhaohai (KI6) (9), Lieque (LU7) (5), Houxi (SI3) (2), Zulinqi (GB41) (2), Gongsun (SP4) (1)
Lower-he-sea point		41	3.83%	3	1.68%	Zusanli (ST36) (30), Yanglingquan (GB34) (9), Weizhong (BL40) (2)
Eight influential points		24	2.24%	6	3.35%	Yanglingquan (GB34) (9), Zhongwan (CV12) (6), Xuanzhong (GB39) (4), Geshu (BL17) (2), Taiyuan (LU9) (2), Danzhong (CV17) (1)
Front-mu point		13	1.21%	4	2.23%	Zhongwan (CV12) (6), Guanyuan (CV4) (5), Danzhong (CV17) (1), Tianshu (ST25) (1)
Xi-cleft point		4	0.37%	3	1.68%	Liangqiu (ST34) (2), Diji (SP8) (1), Jiaoxin (KI8) (1)

#### 3.2.3 Association rules and co-occurrence network analysis

Among the retained rules with 100% confidence, Jiache (ST6) + Lianquan (CV23) → Dicang (ST4) had the highest antecedent support (22.58%) and a lift of 3.35. The next highest-support rules were Yifeng (TE17) + Jinjin (EX-HN12) → Yuye (EX-HN13) and Yifeng (TE17) + Yuye (EX-HN13) → Jinjin (EX-HN12), each with a support of 14.52%, a confidence of 100%, and a lift of 3.02. Xiaguan (ST7) + Lianquan (CV23) → Jiache (ST6) showed the highest lift among all retained rules (3.65), indicating a particularly specific co-selection pattern that is unlikely to be explained by the baseline prevalence of Jiache alone. The complete association rule results are presented in Table 3.

**Table 3:** Association rules analysis of high-frequency acupoints.

Association rules	Count*	Support* (%)	Confidence (%)	Lift
Jiache (ST6) + Lianquan (CV23) → Dicang (ST4)	28	22.58	100.00	3.35
Yifeng (TE17) + Jinjin (EX-HN12) → Yuye (EX-HN13)	18	14.52	100.00	3.02
Yifeng (TE17) + Yuye (EX-HN13) → Jinjin (EX-HN12)	18	14.52	100.00	3.02
Xiaguan (ST7) → Dicang (ST4)	16	12.90	100.00	3.35
Xiaguan (ST7) + Jiache (ST6) → Dicang (ST4)	15	12.10	100.00	3.35
Xiaguan (ST7) + Lianquan (CV23) → Jiache (ST6)	14	11.29	100.00	3.65
Xiaguan (ST7) + Lianquan (CV23) → Dicang (ST4)	14	11.29	100.00	3.35
Panglianquan → Lianquan (CV23)	13	10.48	100.00	1.53
Jiache (ST6) + Baihui (GV20) → Dicang (ST4)	13	10.48	100.00	3.35
Jiache (ST6) → Dicang (ST4)	34	27.42	97.06	3.25
Jinjin (EX-HN12) + Lianquan (CV23) → Yuye (EX-HN13)	30	24.19	96.67	2.92

*Count and support refer to the number and proportion of prescriptions containing the antecedent, respectively. Repeated records of the same acupoint within a prescription were counted once in the association rule analysis.

A high-frequency acupoint co-occurrence network was created using Cytoscape 3.9.1, where the larger the node, the higher the frequency of acupoint usage, and the thicker the line, the stronger the co-occurrence. The results showed that Lianquan (CV23)-Fengchi (GB20), Jinjin (EX-HN12)-Yuye (EX-HN13), Lianquan (CV23)-Yuye (EX-HN13), and Jiache (ST6)-Dicang (ST4) are the core acupoints. The association rules are shown in Table 3. The co-occurrence network diagram group is shown in Figure 3.

**Figure 3:**
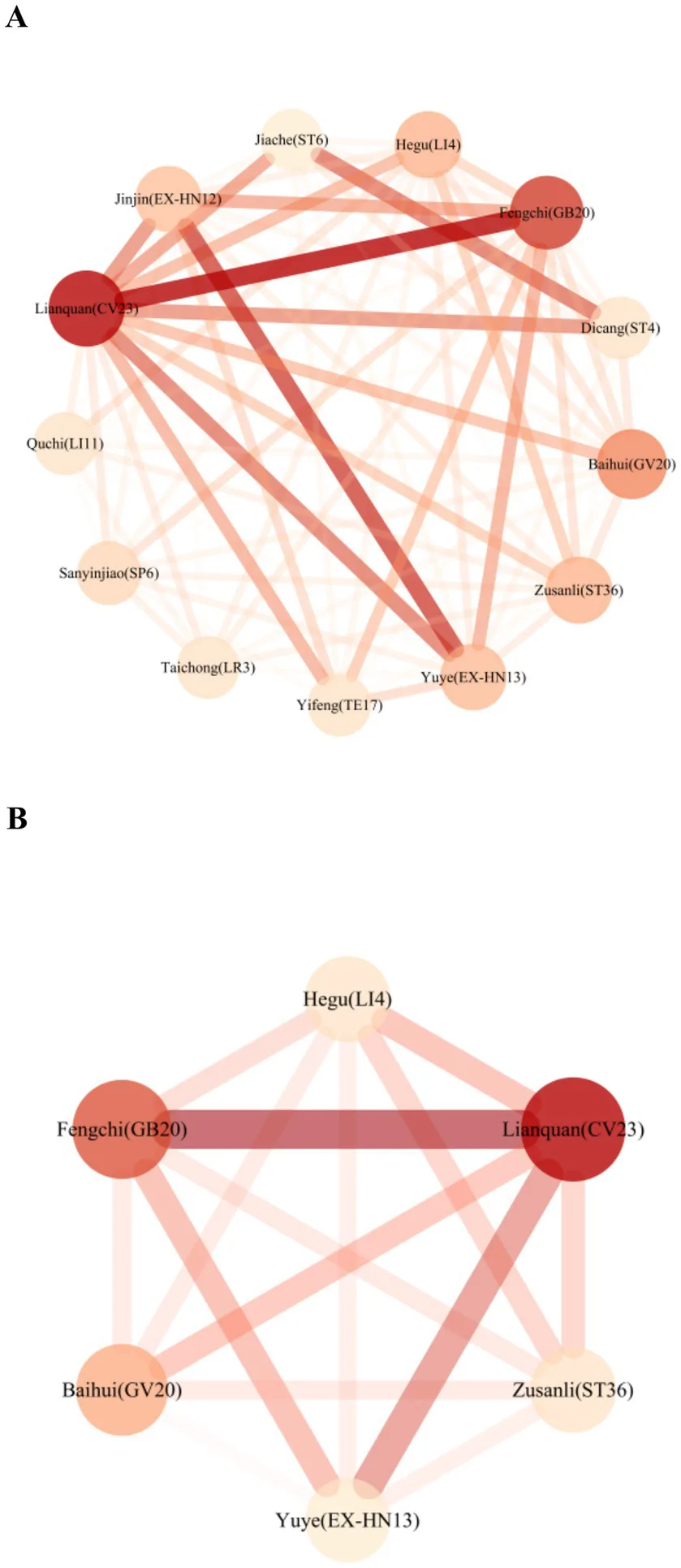
Network diagram of core acupoint prescriptions for the treatment of dysphagia with excessive salivation. **(A)** Connectivity > 15. **(B)** Connectivity > 30.

## 4 Discussion

Dysphagia severely impairs patients’ quality of life and is often accompanied by sialorrhea. Management remains challenging and depends on the underlying cause. Acupuncture is a traditional medical intervention that has been shown to modulate multiple molecular and neural pathways, thereby enhancing orofacial muscular contraction and reducing muscle atrophy (13). There is evidence that acupuncture can be beneficial in cases of both true and pseudobulbar palsy resulting from neurodegenerative disorders, cerebral infarction and cerebral palsy (14).

In this study, the most frequently selected acupoints for the treatment of dysphagia accompanied by sialorrhea were Lianquan (CV23) (82 times, 66.13%), Fengchi (GB20) (59 times, 47.58%), Jinjin (EX-HN12) (42 times, 33.87%), and Baihui (GV20) (38 times, 30.65%). A total of 179 acupoints identified across 124 studies were subjected to association rule analysis. The analysis revealed strong associations among several acupoint combinations, particularly between Dicang (ST4), Jiache (ST6), and Lianquan (CV23), as well as between Yuye (EX-HN13), Yifeng (TE17), and Jinjin (EX-HN12).

Based on the classification of acupoint types, the Five-Shu points are the most frequently applied, followed by the Yuan-primary and Luo-connecting points. From the perspective of meridian theory, the therapeutic rationale for acupuncture in the treatment of dysphagia with sialorrhea accords with the traditional concept of “treating flaccidity by selecting points on the Yangming Meridian” (15). The frequent involvement of the Foot-Yangming Stomach Meridian in the present analysis was largely attributable to the repeated selection of Dicang (ST4), Jiache (ST6), and Zusanli (ST36), reflecting the combined use of local orofacial and distal acupoints.

In clinical practice, acupoints located in close proximity to the affected regions were predominantly selected, especially Lianquan (CV23), Panglianquan, Jiache (ST6), Dicang (ST4), Jinjin (EX-HN12), Yuye (EX-HN13), and Yifeng (TE17), have demonstrated therapeutic efficacy in managing dysphagia with excessive salivation. The most commonly used distal acupuncture points are Zusanli (ST36), Sanyinjiao (SP6), Quchi (LI11), Taichong (LR3) and Hegu (LI4). Using the Apriori algorithm for acupoint association analysis, the results revealed that Lianquan (CV23), Fengchi (GB20), Yuye (EX-HN13), Baihui (GV20), Hegu (LI4), and Zusanli (ST36) were closely interconnected, forming the fundamental components of the acupuncture prescription.

Lift changed the interpretation of several apparently strong rules. Panglianquan → Lianquan (CV23) had a confidence of 100% but a lift of only 1.53. Because Lianquan was already included in nearly two-thirds of the prescriptions, this rule provides only moderate evidence that the two acupoints were preferentially selected together. A stronger pattern was observed for Xiaguan (ST7) + Lianquan (CV23) → Jiache (ST6), which had the highest lift of 3.65. All 14 prescriptions containing both Xiaguan and Lianquan also included Jiache, although Jiache appeared in only 34 prescriptions overall. This pattern is more consistent with a specific orofacial-submandibular combination. Several rules with Dicang (ST4) as the consequent had identical or similar lift values because they shared the same consequent prevalence and comparable confidence; this numerical similarity does not imply equivalent clinical effects. The rules involving Yifeng (TE17), Jinjin (EX-HN12), and Yuye (EX-HN13) also had lift values of approximately 3, making their co-occurrence less readily attributable to frequent acupoint use alone. Their combination may reflect a local strategy involving posterior-auricular and sublingual points for lingual and oropharyngeal dysfunction. These patterns identify combinations that merit further investigation, but they describe prescription behavior rather than proven therapeutic synergy.

Local acupoints in the orofacial, submental, and upper cervical regions are frequently selected because of their proximity to structures involved in oral and pharyngeal swallowing. Lianquan (CV23), located in the submental region above the hyoid bone, has been reported to activate the cortex–parabrachial nucleus-nucleus tractus solitarius circuit and thereby promote swallowing-related neural plasticity (16, 17). Acupuncture at Fengchi (GB20) immediately increased the surface electromyographic amplitudes of the submental and infrahyoid muscles, shortened geniohyoid movement time, and increased geniohyoid displacement (18). A small clinical study reported that an acupuncture protocol including Lianquan (CV23) and Fengchi (GB20) improved FEES-assessed pharyngeal sensory and motor function and reduced secretion accumulation, aspiration, and pharyngeal residue (19). However, without single-acupoint control groups, it remains unclear whether combining Lianquan (CV23) and Fengchi (GB20) provides any additional benefit. Dicang (ST4) and Jiache (ST6), both located on the Foot-Yangming Stomach Meridian, were frequently paired in the prescriptions analyzed in this study. A retrospective clinical study also included both points in an acupuncture regimen for post-stroke dysphagia, with transverse needling from Dicang toward Jiache (20). Their locations in the perioral and masseteric regions, respectively, provide a plausible anatomical basis for complementary effects on lip closure and mandibular control (21, 22). Jinjin (EX-HN12) and Yuye (EX-HN13), two extra points located on either side of the lingual frenulum, are commonly used together (23). A clinical study reported that adding pricking at Jinjin and Yuye to body acupuncture resulted in a higher overall response rate than body acupuncture alone, suggesting an additional clinical benefit from including this acupoint pair (24). Yifeng (TE17), located posterior to the earlobe between the mastoid process and the mandible, was frequently selected in the included prescriptions, but direct mechanistic evidence linking this acupoint to swallowing or salivary control remains limited (25). The available evidence supports potentially complementary roles for these acupoint pairs but does not establish synergistic efficacy. Demonstrating synergy will require studies comparing each acupoint alone with their combination and determining whether the combined effect exceeds the expected additive effect.

Acupuncture at Zusanli (ST36) has been shown to modulate vagal tone, which may contribute to improved gastrointestinal and oropharyngeal function. Its anti-inflammatory and antioxidant effects have also been suggested to provide neural protection (26). Acupuncture at ST36 may modulate autonomic activity, which has been implicated in the neural regulation of salivary secretion (27). Stimulation of Quchi (LI11) has pronounced anti-inflammatory and immunomodulatory effects, downregulating central signaling pathways and alleviating neuroinflammation while improving medullary function (28). Laser acupuncture at Hegu (LI4) and Shousanli (LI10) altered corticospinal and intracortical excitability, although whether these effects translate into improved swallowing remains unknown (29, 30). Mechanistic evidence for individual acupoints remains limited. Reviews of acupuncture in stroke rehabilitation have described potential effects on cerebral blood flow, apoptosis, neurochemical signaling, and synaptic plasticity; whether these mechanisms contribute specifically to dysphagia or salivary control remains uncertain (31, 32). Previous studies have suggested that acupuncture may improve dysphagia through modulation of neural function and inflammatory responses. Swallowing is regulated by a complex brainstem-cortical network, including pathways such as the primary motor cortex (M1)-parabrachial nucleus (PBN)-nucleus tractus solitarius (NTS) circuit (16, 33). Acupuncture stimulation may influence neural activity within this network, thereby improving swallowing coordination. Distal acupoints such as Hegu (LI4), Zusanli (ST36), Sanyinjiao (SP6), Quchi (LI11), and Taichong (LR3) were frequently selected; however, direct evidence linking each point to specific swallowing- or salivation-related mechanisms remains limited.

These findings suggest that acupuncture may exert therapeutic effects through coordinated regulation of neural activity and inflammatory processes. However, the underlying mechanisms remain to be further clarified by future studies.

## 5 Limitations

This study has several limitations. First, the number of included studies was relatively limited, and most were of moderate methodological quality, with inconsistencies in diagnostic criteria, intervention protocols, and outcome measures across studies. Second, the analysis did not distinguish between true bulbar palsy and pseudobulbar palsy, which may have introduced heterogeneity in the clinical features and therapeutic responses of patients with dysphagia. Third, the use of secondary data extracted from published literature limited the ability to assess individualized treatment parameters such as needling depth, stimulation intensity, and duration. In addition, publication bias cannot be completely ruled out, as most included studies originated from Chinese databases. Future research should incorporate large-scale, multicenter randomized controlled trials with standardized evaluation systems to verify and refine the acupoint selection patterns identified through data mining.

## 6 Conclusion

In summary, this data-mining analysis revealed that acupuncture for dysphagia accompanied by sialorrhea predominantly involves the Foot-Yangming Stomach Meridian, the Governor Vessel, and the Conception Vessel. Among the acupoints, Lianquan (CV23), Fengchi (GB20), Yuye (EX-HN13), Baihui (GV20), Hegu (LI4), and Zusanli (ST36) were identified as core points forming the fundamental prescription network. From a clinical perspective, these findings support a provisional local–distal acupoint selection framework rather than a fixed prescription. These findings provide a quantitative description of current acupuncture prescribing practices for dysphagia with sialorrhea and identify frequently used acupoints and stable co-occurrence pairs that merit further clinical validation. However, several clinically relevant parameters—including needling depth and direction, stimulation intensity, retention time, treatment frequency, and the choice between manual and electroacupuncture-could not be reliably extracted from the included retrospective literature. Future large-sample, multicenter randomized controlled trials with standardized evaluation frameworks are needed to validate and refine these patterns before they can inform clinical protocol development.

## Data Availability

The original contributions presented in the study are included in the article/Supplementary material, further inquiries can be directed to the corresponding authors.

## References

[1] Cichero JA Lam P Steele CM Hanson B Chen J Dantas RO . Development of international terminology and definitions for texture-modified foods and thickened fluids used in dysphagia management: the IDDSI framework. Dysphagia. (2017) 32:293–314. doi: 10.1007/s00455-016-9758-y, 27913916 PMC5380696

[2] Thiyagalingam S Kulinski AE Thorsteinsdottir B Shindelar KL Takahashi PY. Dysphagia in older adults. Mayo Clin Proc. (2021) 96:488–97. doi: 10.1016/j.mayocp.2020.08.001, 33549267

[3] NHS. Guidance on options for the pharmacological management of hypersalivation in adult neurology patients (patients aged 18 and over). London: South East London Area Prescribing Committee (2021). Available online at: https://www.selondonics.org/wp-content/uploads/dlm_uploads/2022/11/Hypersalivation-management-options-FINAL-September-2019.pdf (Accessed September 01, 2021).

[4] Paine CC 2nd Snider JW 3rd. When saliva becomes a problem: the challenges and palliative care for patients with sialorrhea. Ann Palliat Med. (2020) 9:1333–9. doi: 10.21037/apm.2020.02.34 32279509

[5] Azer SA Kanugula AK Kshirsagar RK. Dysphagia. Treasure Island (FL): StatPearls Publishing (2023). 32644600

[6] Li L Xu F Yang S Kuang P Ding H Huang M . Tongue acupuncture for the treatment of post-stroke dysphagia: a meta-analysis of randomized controlled trials. Front Neurosci. (2023) 17:1124064. doi: 10.3389/fnins.2023.1124064, 37304013 PMC10247969

[7] Liu R Liang L Huang H Zeng Z Sun J. Rehabilitation effects of acupuncture on the diaphragmatic dysfunction in respiratory insufficiency: a systematic review and meta-analysis. Complement Ther Med. (2024) 87:103105. doi: 10.1016/j.ctim.2024.103105, 39490936

[8] Zhu PY Guan SY Liu JT Chen D Gao WB. Gao Wei-bin's clinical experience in treatment of medulla oblongata paralysis with nape acupuncture. Zhongguo Zhen Jiu. (2022) 42:1285–9. doi: 10.13703/j.0255-2930.20220101-k0001 36397227

[9] Xu F Zhang Y Su X Dai F Ye Y Ling M . Effects of acupuncture combined with conventional rehabilitation training for patients with post-stroke dysphagia: a randomized controlled trial. J Multidiscip Healthc. (2025) 8:3139–52. doi: 10.2147/jmdh.S526827, 40486255 PMC12143297

[10] Wang P Ma X Huang J Li J Ma L Xu D . Effect of acupuncture treatment on dysphagia caused by pseudobulbar paralysis after stroke: a systematic review and meta-analysis. Ann Palliat Med. (2022) 11:2257–64. doi: 10.21037/apm-21-3551, 35400158

[11] State Administration of Traditional Chinese Medicine. State Administration of Traditional Chinese Medicine. Nomenclature and Location of extra Points in common use. Beijing: State Administration for Market Regulation, China National Standardization Administration Committee GB/T 40997-2021 (2021).

[12] State Administration of Traditional Chinese Medicine. Nomenclature and Location of meridian Points. Beijing: State Administration for Market Regulation, China National Standardization Administration Committee (2021).

[13] Zhang S Liang B Tang Q Gao W Li H Wu M . Therapeutic effect of acupuncture on post-stroke dysphagia: a multicenter, randomized controlled trial. Complement Ther Med. (2025) 92:103200. doi: 10.1016/j.ctim.2025.103200, 40545007

[14] Li H Li J Wang X Zhang Z. A systematic review and meta-analysis of acupuncture in aspiration caused by post-stroke dysphagia. Front Neurol. (2024) 15:1305056. doi: 10.3389/fneur.2024.1305056, 38915795 PMC11194430

[15] Hou TS Feng M Wu QF. The analysis of "treatment for flaccidity aims at YangMing meridian" and clinical application of acupuncture. Gansu J Tradit Chin Med. (2011) 24:3–4.

[16] Yao L Ye Q Liu Y Yao S Yuan S Xu Q . Electroacupuncture improves swallowing function in a post-stroke dysphagia mouse model by activating the motor cortex inputs to the nucleus tractus solitarii through the parabrachial nuclei. Nat Commun. (2023) 14:810. doi: 10.1038/s41467-023-36448-6, 36781899 PMC9925820

[17] Qin L Zhang XP Yang XC Cui CH Shi J Jia CS. Deep acupuncture of Lianquan (CV23) and Yifeng (TE17) in combination with conventional acupuncture of other acupoints is superior to swallowing rehabilitation training in improving post-stroke dysphagia in apoplexy patients. Zhen Ci Yan Jiu. (2019) 44:144–7. doi: 10.13702/j.1000-0607.180018, 30945493

[18] Gao JX Zhou HF. Therapeutic effect of nape cluster acupuncture combined with swallowing function training on post-stroke dysphagia. Zhongguo Zhen Jiu. (2020) 40:586–90. doi: 10.13703/j.0255-2930.20190609-k0001, 32538006

[19] Qin Y Lin SX Chen XK Chen WY Chen HX Lu LM. Clinical effect of acupuncture on dysphagia following traumatic brain injury based on fiberoptic endoscopic evaluation of swallowing. Zhen Ci Yan Jiu. (2026) 51:358–65. doi: 10.13702/j.1000-0607.20250015, 41839730

[20] Wang Q Ke X. Application of dietary training combined with acupuncture based on syndrome differentiation for treating dysphagia in stroke rehabilitation patients. Arch Med Sci. (2026) 22:1420–32. doi: 10.5114/aoms/204368, 42540604 PMC13426157

[21] Zhong L Wang J Li F Bao X Liu H Wang P. The effectiveness of acupuncture for dysphagia after stroke: a systematic review and Meta-analysis. Evid Based Complement Alternat Med. (2021) 2021:1–18. doi: 10.1155/2021/8837625, 33531922 PMC7837771

[22] Mohamad N de Oliveira-Souza AIS de Castro-Carletti EM Müggenborg F Dennett L McNeely ML . The effectiveness of different types of acupuncture to reduce symptoms and disability for patients with orofacial pain. A systematic review and meta-analysis. Disabil Rehabil. (2024) 46:5700–16. doi: 10.1080/09638288.2024.2310766, 38357796

[23] Yan J Li R Jiang Y Chen Z Yan S He J . Case of multiple cranial nerve injury. Zhongguo Zhen Jiu. (2025) 45:742–4. doi: 10.13703/j.0255-2930.20250218-k0004 40518776

[24] Zhang H Jiang YJ Wei QL Dong XL. Efficacy observation on body acupuncture and point pricking at Jinjin (EX-HN12) and Yuye (EX-HN13) for post-stroke dysphagia. Zhongguo Zhen Jiu. (2013) 33:11–3.

[25] Yuan S Qiu B Liang Y Deng B Xu J Tang X . Role of TRPV1 in electroacupuncture-mediated signal to the primary sensory cortex during regulation of the swallowing function. CNS Neurosci Ther. (2024) 30:e14457. doi: 10.1111/cns.14457, 37718934 PMC10916430

[26] Yue G Yuan X Ge X Du G. Effect of electroacupuncture at different acupoints in treating rapid gastric emptying: Zusanli (ST36) plus Zhongwan (CV12) versus Zhongwan (CV12) alone. J Tradit Chin Med. (2021) 41:326–30. 33825414

[27] Liu S Wang Z Su Y Qi L Yang W Fu M . A neuroanatomical basis for electroacupuncture to drive the vagal-adrenal axis. Nature. (2021) 598:641–5. doi: 10.1038/s41586-021-04001-4, 34646018 PMC9178665

[28] Lan L Tao J Chen A Xie G Huang J Lin J . Electroacupuncture exerts anti-inflammatory effects in cerebral ischemia-reperfusion injured rats via suppression of the TLR4/NF-κB pathway. Int J Mol Med. (2013) 31:75–80. doi: 10.3892/ijmm.2012.1184, 23165960

[29] Liu R Zheng Z Moe AAK Zoghi M Jaberzadeh S. Laser acupuncture improves cortical excitability and behavioural performance in healthy individuals: a randomized controlled trial. BMC Complement Med Ther. (2026) 26:59. doi: 10.1186/s12906-026-05259-9, 41566337 PMC12905872

[30] Cheng I Li WQ Hamdy S Michou E Huckabee ML Tomsen N . Brainstem stroke and dysphagia treatment: a narrative review on the role of neuromodulation, skill-based swallowing training and transient receptor potential agonists. Audiol Res. (2025) 15:156. doi: 10.3390/audiolres15060156, 41283499 PMC12641870

[31] Chavez LM Huang SS MacDonald I Lin JG Lee YC Chen YH. Mechanisms of acupuncture therapy in ischemic stroke rehabilitation: a literature review of basic studies. Int J Mol Sci. (2017) 18:2270. doi: 10.3390/ijms18112270, 29143805 PMC5713240

[32] Zhuo B Deng S Li B Zhu W Zhang M Qin C . Possible effects of acupuncture in Poststroke aphasia. Behav Neurol. (2023) 2023:1–18. doi: 10.1155/2023/9445381, 37091130 PMC10115536

[33] Pitts T Huff A Reed M Iceman K Mellen N. Evidence of intermediate reticular formation involvement in swallow pattern generation, recorded optically in the neonate rat sagittally sectioned hindbrain. J Neurophysiol. (2021) 125:993–1005. doi: 10.1152/jn.00623.2020, 33566745 PMC8282227

