# Supplementary material for: Data mining of acupoint selection and combination patterns for dysphagia with sialorrhea: a literature-based study
Source: Front Neurol. 2026 Sep 22;17:1849484. doi: 10.3389/fneur.2026.1849484 (PMC13638387; doi:10.3389/fneur.2026.1849484)
Supplement: Supplementary file 1 [file Data_Sheet_1.ZIP › search strategy for literature retrieval.docx]

**[CNKI]**
TKA=(acupuncture OR needling OR acupoint OR meridian OR scalp acupuncture OR abdominal acupuncture OR warm needling OR fire needling OR electroacupuncture) AND TKA=(swallowing OR dysphagia OR deglutition disorders) AND FT=(sialorrhea OR hypersalivation)
Total: 705 records

**[Wanfang]**
Topic: (acupuncture OR needling OR acupoint OR meridian OR scalp acupuncture OR abdominal acupuncture OR fire needling OR electroacupuncture OR warm needling) AND Topic: (swallowing OR deglutition disorders OR dysphagia) AND Full-text: (sialorrhea OR hypersalivation)
Total: 104 records (7 full texts not available)

**[CQVIP]**
M=(acupuncture OR needling OR acupoint OR meridian OR scalp acupuncture OR abdominal acupuncture OR warm needling OR fire needling OR electroacupuncture) AND M=(swallowing OR dysphagia OR deglutition disorders) AND U=(sialorrhea OR hypersalivation)
Total: 26 records

**[PubMed]**

("Acupuncture therapy" OR "acupuncture" OR "acupoint" OR "meridian" OR "Fire Needle" OR "Scalp Acupuncture" OR "Abdominal Acupuncture" OR "Electroacupuncture" OR "Needle warming moxibustion") AND ("Deglutition Disorder" OR "Disorders, Deglutition" OR "Dysphagia" OR "Swallowing Disorders" OR "Swallowing Disorder" OR "Oropharyngeal Dysphagia" OR "Dysphagia, Oropharyngeal" OR "Esophageal Dysphagia" OR "Dysphagia, Esophageal")
[236 records]

**[Sinomed]**
("acupuncture"[All Fields] OR "needling"[All Fields] OR "acupoint"[All Fields] OR "scalp acupuncture"[All Fields] OR "fire needling"[All Fields] OR "abdominal acupuncture"[All Fields] OR "electroacupuncture"[Mesh] OR "warm needling"[Mesh] OR "meridian"[Mesh]) AND ("swallowing"[All Fields] OR "deglutition disorders"[All Fields] OR "dysphagia"[All Fields]) AND ("sialorrhea"[All Fields] OR "hypersalivation"[All Fields])
[64 records]

**[Web of Science]** AND 【 TS=(“Sialorrhea” OR “Drooloing” OR “Hypersalivation”)】
TS=("Acupuncture therapy" OR "acupuncture" OR "acupoint" OR "meridian" OR "Fire Needle" OR "Scalp Acupuncture" OR "Abdominal Acupuncture" OR "Electroacupuncture" OR "Needle warming moxibustion") AND TS=("Deglutition Disorder" OR "Disorders, Deglutition" OR "Dysphagia" OR "Swallowing Disorders" OR "Swallowing Disorder" OR "Oropharyngeal Dysphagia" OR "Dysphagia, Oropharyngeal" OR "Esophageal Dysphagia" OR "Dysphagia, Esophageal")
[271 records]
AND TS=("Sialorrhea" OR "Drooloing" OR "Hypersalivation")

**[ScienceDirect]**
("Acupuncture therapy" OR "acupuncture" OR "acupoint" OR "meridian") AND ("Deglutition Disorder" OR "Dysphagia") AND ("Sialorrhea" OR "Drooloing" OR "Hypersalivation")
[107 records]

**[Cochrane Library]**
("Acupuncture therapy" OR "acupuncture" OR "acupoint" OR "meridian") in Title Abstract Keyword AND ("Deglutition Disorder" OR "Dysphagia") in Title Abstract Keyword AND ("Sialorrhea" OR "Drooloing" OR "Hypersalivation") in All Text
[2 records]
